# Curiosities of Native Treatment in Fiji

**Published:** 1919-02-08

**Authors:** T. R. St.-Johnston

**Affiliations:** Colonial Civil Service.


### CURIOSITIES OF NATIVE TREATMENT IN FIJI.

By T. R. ST.-JOHNSTON, M.R.C.S., L.R.C.P., Colonial Civil Service.

The "Tokelau ringworm" (*Tinea imbricata*), with which I have seen scores of natives literally covered from head to foot, is said not to have been in Fiji for more than a few generations; probably, as its name implies, being first imported from the Tokelau or Line Islands in comparatively recent times. But already the natives have a remedy for it, a strong blistering fluid from the juice of the "Kau Karo" (*Oncocarpus vitiensis*). This is a very powerful irritant, and if too much is used causes a pustular rash, but probably in the process settles the "trichophyton." If the mainland people had been more cleanly and had destroyed infected clothing and mats the disease might have been checked by their own remedies, but as it was it spread to such an extent that special Government measures had to be taken against it, and at last it is being overcome, chiefly by sulphur vapour, etc. It is noteworthy that in the Eastern Islands, where the people go in for much sea-bathing and shore-fishing, and where they afterwards anoint themselves liberally with pure coconut oil, this disease is practically unknown.

For elephantiasis and bouts of "Waqaqa" (filarial fever) the natives admit they have no real cure, but some good effects having been obtained with salvarsan the filarial patients are now almost as anxious to have it tried on them as the sufferers from yaws are. The faith of the native in salvarsan ("Wai-ni-cula," literally "the medicine-that-is-injected") is most encouraging where yaws is concerned, for with it they can see the big ulcers of tertiary yaws almost healing up before their eyes. In the early days they considered that it would be better for every child to acquire yaws and get it over, in much the same way as the lower classes at home used to regard measles and to give their children even the "cow-pox." Occasionally the natives would put a poultice of "lewe-ni-sau" leaves on such of the yaws papules as had run together into a mass, but usually they were just left to the flies to browse upon—a very ugly sight! Conditions are now much improved, and it is hoped that before many years this disease may be a comparatively rare one. It was responsible for many other troubles, amongst which lunacy may be numbered; as it is undoubtedly a cause of G.P.I. with these natives (among whom syphilis is unknown), just as syphilis is a big causative agent of it in other parts of the world. The enlarged ideas of a Fijian G.P.I. are, as may be imagined, sometimes very amusing. I remember once examining a man called Tevita, who, incidentally, had been a native preacher. He told me, among other things, that he had just returned from a visit to heaven. I asked him what it was like. He replied "Glorious. There were sing-songs every evening, and we actually had mutton for dinner every day"!

Leprosy is a disease that is unfortunately not at all rare among them, and cases of it are now by law segregated on a very modern leper station, an island, where the latest methods of treatment are applied. But in the old-days the natives had a very barbarous cure, or alleged cure, for it, as follows:—The wretched sufferer was, generally of his own choice, bound hand and foot, and, suspended in a hut, literally smoked alive over a fire of "Sinu Gaga" wood (*Excoecaria Agallocha*), the acrid fumes of which peeled off the surface lesions of his horrible complaint, and if he lived through it gave him, it was stated, a new lease of life. It seems very doubtful, however, if a disease caused by general bacillary infection could really be checked in this manner.

Another remedy the natives used was an infusion of the "Totodra" leaves, but I don't think they ever honestly regarded leprosy as a curable disease; and even in their primitive conditions they had usually insisted on lepers being segregated in huts by themselves in "the bush." In fact I remember once the natives of Boitaci driving back to his hut with sticks a leper who insisted on coming into their town. Certain well-known stones were supposed to have inherent properties of causing leprosy, but that fringes on another topic, magic and folk-lore, which, though interesting, is not within the scope of this article. Before closing I must mention two other complaints that trouble the more primitive Fijians a good deal. One is a peculiar scaling of the skin found in chronic "Kava" topers ("Kava" or "Yagona" being that well-known drink of the South Seas made from the crushed root of the *Piper mythisticum*). This drink, if taken to excess, leaves the head perfectly clear, but intoxicates the legs; so that the midnight roysterers are unable to move —though it has to be a very heavy bout that brings about such a state of affairs.

The other is no less than the common trouble of lice, which find a peculiarly heaven-sent home in the luxuriant up-standing hair of the native; but it is a trouble generally of short-lived duration, as the Fijian really compares very favourably in cleanliness with natives from any other part of the world, and at the first sign of this invasion he promptly steeps his hair in a strong plaster of coral-lime. This gives him a peculiar appearance for a few hours, but quickly has the desired effect, and after several applications a really fine shade of golden-auburn hair is obtained. There are equally interesting methods of surgical and obstetrical treatment among these South Sea Islanders, but space is limited, and I must for the present conclude.

### SURGICAL.

I have dealt above with questions of medical treatment as practised by the natives of Fiji, and I now propose to describe some of the curious methods of surgery that used to be in vogue amongst them, and that are still to be found in the more remote villages. Needless to say, surgical asepsis as we know it is unknown to

☛ The first article appeared in the "Hospital" of January 18.

**Curiosities of Native Treatment in Fiji**—(*cont*.).

them (I exclude, of course, the trained native medical students from the Government Hospital, who are taught just the same methods as any medical student at home.) Yet, despite the lack of asepsis, so pure is the atmosphere and so little is the general contamination in this country far removed from overcrowded cities that some really hair-raising exploits in surgery have often been done with a success that justified the venture.

In the early days of the British Government in the colony there was still a considerable amount of tribal warfare going on (in addition to an actual "war" on a small scale that was brought about by an uprising of the heathen cannibals in the mountains), and the wounds to be treated were mostly caused by spears—long, barbed, vicious-looking things—and by club blows on limbs and heads. If the latter, there was little need for a surgeon!

Old "Sniders," equally dangerous to friend or foe, caused when stuffed with scrap-iron very similar wounds to those of modern explosive shells—a sorry testimony to the advance of civilisation—but the feelings of a Fijian "Tommy" towards his R.M.O. were hardly the same as those now held by Thomas Atkins in France. Sir William MacGregor, late Governor of Queensland, was a medical officer in the Fiji Service at the time I speak of, and on successfully amputating the leg of one of the Fijian soldiers, the latter turned round to him and said, with perfect sincerity and faith in his request, "Now, sir, according to our custom it is your duty to keep me and support me for the rest of my life!"

But amputations by the natives themselves were very seldom done, the dread of the knife and of losing a portion of one's physical entity being an idea that probably sprung from their Eastern ancestry. The name for a surgeon was "Matai-ni-mate" (literally the Carpenter of Sickness), and a very good name too, when one comes to consider it. Moreover, the carpenters were a guild unto themselves, the most venerated and respected of all being regarded more as chiefs; in fact the chiefs, if clever enough, often became carpenters. A carpenter had need to be skilful in those days, to make beautifully smoothed wooden bowls, canoes, etc., with a stone knife or adze, instruments which I have myself seen still in use in the mountain districts. A stone knife for coarse work, a scalpel of split bamboo with its razor-like edge for skin and soft parts, and a clever use of nooses for removing foreign bodies, were the chief implements of a native surgeon; while skilful fingers helped to make up for lack of the many patent appliances that are used by the Harley Street man at home. Speaking of the "Matai-ni-mate," the word "mate" is doubly curious, firstly inasmuch as it is universal throughout the Pacific and extends as far as the Malay Peninsula (one more confirmation of the theory that the Fijians and Maoris sprang from the Malay regions); and secondly as it means either disease or even death, the two ideas being curiously intermingled in the native's mind. Thus when a very sick or very aged person was apparently beyond all aid he or she was considered by the natives as "dead," and not so very long ago actually buried alive (and not unwillingly either, it being ancient custom). This practice was of course stopped when the British Government took over the colony, but traces of it still remain in the wrapping in the grave-clothes and preparing for burial of such people while still alive. Only recently I heard that an old chief was very ill, and on going to see him found him all laid out for burial in sheets of "tappa" (native cloth prepared from the bark of the Broussonetia tree). However, I saw that he was not really *in extremis*, and gave him some simple medicines, resulting in his being up and energetically digging in his vegetable garden on my next visit!

Some unusual accident cases that the native surgeon has often to deal with are falls from the top of a cocoanut tree (a favourite way of committing suicide), rips from the tusk of a wild boar, and savage slashes by the "oga" fish at the abdomen of women engaged in shallow-water fishing. The "oga" is a variety of small sword-fish, and can inflict a nasty penetrating wound, more dangerous really than the bite of a shark.

To show how different the conception of surgical sepsis may very well be in an English manufacturing city and out in these South Sea Islands, I may mention a case I was once called in to see up-country in a native village. A small boy about ten years of age was whittling a stick with a broad-bladed, rather rusty knife, when some of his playmates began laughingly to chase him. As he was running, still clasping his knife, he tripped over the root of a bread-fruit tree, and fell upon the point of the blade, which penetrated his chest to a depth of some four inches, just missing his heart. It was some hours before I reached him, and then I had nothing but a pocket-case and some compressed gauze dressings with me. I packed the wound, in which lung substance could be seen bubbling and sucking air, and bandaged him up and left directions for him to be brought in by a canoe (a day-and-a-half journey) to the hospital. However, a gale sprang up and he was not brought in, and when I went out on the third day to see why he had not been moved yet, I found him walking quietly about, and a week later he was playing games with his friends as if nothing had ever happened. On another occasion I received news that an accidental shooting case had occurred on another island, two days' journey across a rough and open sea. On arrival I found that the schoolmaster's cook had been shot in the head by a Solomon Islander who had pointed a Winchester at him in fun. I probed for the bullet, which had penetrated deeply, under an anæsthetic but without success, and as there were no untoward symptoms I thought it advisable to leave well alone, and in a few days' time I found the man taking a busman's holiday by cheerfully cooking turtle, against all regulations, for the other hospital patients!

